# Views and Experiences of Health Service Users in Greece During 2012–2018

**DOI:** 10.7759/cureus.32769

**Published:** 2022-12-21

**Authors:** Alexandra Vlassi, Georgios Tzikos, Magda Pappa, Theodoros Dardavesis, Theodosios Papavramidis

**Affiliations:** 1 Laboratory of Hygiene, Social & Preventive Medicine and Medical Statistics, Department of Medicine, School of Health Sciences, Aristotle University of Thessaloniki, Thessaloniki, GRC; 2 1st Propedeutic Department of Surgery, American Hellenic Educational Progressive Association (AHEPA) University Hospital of Thessaloniki, Thessaloniki, GRC

**Keywords:** qualitative research, public health, primary health care, health care reform, greece

## Abstract

During the period of 2012-2018, a series of reforms took place in the Greek health care system. These reforms focused on both the health expenditure and the efficiency and quality of health services provided. In this qualitative study, we examined whether these improvements were visible to health service users based on their personal experiences with the health care system, using thematic content analysis to evaluate the research data. The results indicated that the reforms did not meet the original objectives in several fields such as primary health care, health expenditure streamlining, health service affordability, and confidence in the health care system and professionals. Further interventions are required to improve the status of the Greek health care system.

## Introduction

Between 2012 and 2018, a series of reforms to the Greek health care system were adopted, focusing on health expenditure, and efficiency and quality of health services provided. To this day, few studies have analyzed the effectiveness of these reforms. In 2018, the Organization for Economic Co-Operation and Development study evaluates, among others, the anti-corruption reforms in the health care sector (March to August 2017) [1]. The results show that the lack of resources, irregularities, and corruption in health care expenditure management greatly affect sector functioning and its public image. Under these circumstances, the need for access to treatment and timely and adequate service provision leads to the development of small-scale corruption or the corrupt practice of the “envelope” (paying an extra amount of money to doctors working in public hospitals to ensure appropriate treatment). Participants identified cases of outright corruption involving the pharmaceutical industry and politicians, highlighting the weaknesses of health care and governance structures and their impact on service provision.

Tsountas et al. proceeded to a mapping of the Greek health care structures and the health status of the Greek population [2]. According to the annual evaluation of health systems of 45 countries by the Euro Health Consumer Index, Greece slipped down in rankings; specifically, in 2015 Greece was ranked 28th with 577 points (on a scale of 1000), while in 2012, 2013, and 2014 it was ranked 22nd, 25th, and 28th, respectively. The researchers also point out that the financial crisis has forced citizens to change their attitudes toward receiving health services and to turn to public health structures more often to reduce their expenses, while many patients avoid receiving health services because they cannot afford them.

In 2013, Niakas reported that for the health care reforms to be effective, a decentralized administrative and organizational structure should be created, and the provision of services should be adapted to the conditions of each region (urban/rural areas, islands, and remote regions) to serve the insured citizens [3]. According to the study, decision-makers should consider a reform based on the principles of equality and efficiency, including the participation of both representatives of the insured citizens and the state in the administration, in combination with a wide range of options based on the prevailing conditions of service provision (medical staff number, need to attract health care workers in remote areas, etc.) and the behaviors and habits observed in the Greek population.

This study aimed to examine whether the intended improvements in certain health care system fields are in line with the views and personal perspectives and experiences of patients and users of health services in general. Additionally, we aimed to identify the factors underpinning the satisfaction or dissatisfaction of health service users through the evaluation of their reports. Therefore, official data from the Ministry of Health regarding cost reduction, enhancement of primary health care, improvement of the effectiveness of tertiary care, and access to health care services were compared to assessments provided by health service users.

## Materials and methods

We chose to conduct qualitative research to ensure the normal and effortless flow of information from the participants in the research [4]. This provided the opportunity to examine the perceptions and opinions of the participants and to identify and analyze their experiences, as well as the variables that have influenced and shaped their views [5]. The study was approved by the university’s editorial board, and all the participants provided written informed consent.

We decided to include any patient older than 18 years who used any health care service during their outpatient visit or hospitalization in four different tertiary hospitals in Thessaloniki, a city in northern Greece with a population of two million. Their selection was random and lasted from January 1, 2019, to March 1, 2019. Two independent investigators interviewed the participants about their experience regarding health care services and obtained information about their opinion on the newly applied reforms by the government. During the interview, each participant was asked a set of questions based on a total of six thematic axes: (1) Evaluation of primary health care (PHC), (2) Evaluation of measures taken to rationalize health expenditure, (3) Financial accessibility to health services, (4) Personal experiences from the health system, (5) Confidence in the health system, and (6) Trust in health professionals. Next, additional topics were created in each of the six thematic axes. In detail, nine themes and a total of 110 codes were used in the thematic axis of "Evaluation of Primary Health Care (PHC)". The nine subjects recorded: (1) whether the patients have noticed changes in the Primary Health Care (15 codes), (2) their opinion on the treatment of patients with a subcategory for suggestions for improvements (26 codes), (3) their opinion about the electronic file (six codes), (4) what patients have observed about the family doctor with a subcategory for their personal opinion (11 codes), (5) their opinion on emergencies (nine codes), (6) their opinion on on-call staff (11 codes), (7) their opinion on health centers (10 codes), (8) their view of prevention with a subcategory for prevention related to mental health (15 codes), and (9) their opinion on dating (seven codes). In the thematic axis of "Evaluation of measures taken to rationalize health," three themes and a total of 20 codes were used. The three subjects recorded: (1) whether patients have noticed changes based on these measures (six codes), (2) their opinion about generics (seven codes), and (3) their opinion on electronic prescribing (seven codes). In the thematic axis of “Financial accessibility to health services,” four themes and a total of 20 codes were used. The four themes recorded the opinion of health service users regarding: (1) responsiveness to costs (four codes), (2) Greek National Organization for Health Care Services Provision - EOPPY and participation in examinations by individuals (six codes), (3) financial accessibility (5 codes), and (4) the participation rate in drug tests (five codes). In the thematic axis of “Personal experiences from the health system,” 5 themes and a total of 45 codes were used. The five themes captured the view of health service users regarding: (1) the Health Centers of the National Health System - NHS (six codes), (2) the state hospitals of the NHS (16 codes), (3) non-contracted freelance doctors (with patient fee) (five codes), (4) the freelance doctors contracted with the Greek National Organization for Health Care Services Provision- EOPYY (seven codes), and (5) Local Health Unit - TOMY (11 codes). In the thematic axis of “Confidence in the health system,” 2 themes and a total of 12 codes were used. The two2 themes captured the view of health service users regarding: (1) trust in the health system (eight codes) and (2) trust in health professionals (four codes).

Then all the interviews were stored in voice record, which was then analyzed, and the extracted data were recorded in an Excel spreadsheet. Next, these data were included in our qualitative analysis. The analysis was performed by another independent and blind, regarding the aims of the study, investigator to ensure the validity of the data.

To analyze the research data, we employed thematic content analysis, a systematic scientific technique used to reduce the data collected through qualitative techniques. Specifically, the content analysis focuses on identifying common themes in the texts provided for analysis. According to Gibbs, a code identifies and records specific passages in the text that outline the same descriptive or theoretical idea. All textual elements referring to or describing the same idea are encoded under the same name [6]. Coding is a text classification method for identifying a framework of thematic concepts [6]. For data analysis, the principles of thematic analysis were followed as presented by Tsiolis and by Braun and Clarke [7,8]. Various conceptual frameworks developed since the 1960s, such as discrepancy, equity, fulfillment, disconfirmation, and value-expectancy theories focused on the importance of expectations in service users’ satisfaction [9-13]. Additionally, the frameworks for interpreting individual perceptions of service users have followed two different paths in the current period. The SERVPERF (the performance component of the Service Quality scale) approach stems from the notion that service user satisfaction reflects the evaluation of a service provider and states that a performance-only model is more appropriate to measure overall hospital service quality (instrument) [14]. According to the SERVQUAL (Service Quality) approach, known as the “American” perspective, the assessment of patient experience is defined as a form of “attitude,” derived from a comparison between client expectations and perceptions about a firm’s performance [15]. To the best of our knowledge, the instruments employed for thematic content analysis have not been widely utilized in health care related studies in Greece, although they are otherwise extensively used. Therefore, we considered this analysis would greatly contribute to assessing the impact of the Greek health care system reforms on the experience of health service users.

The analysis process was divided into six phases. In the first phase, we focused on reading and understanding the data, and identified the original concepts [8]. All interviews were listened to and notes were taken on the data, each participant's interview sessions were transcribed, and transcripts of the interviews were reviewed for errors or omissions. The first reading also helped the initial identification of the patterns, themes, and codes that would arise [7,8]. Additionally, the NVivo 12 pro software was used to analyze, group, and encode large volumes of quality data [16]. Copies of the recorded interviews were uploaded to the program, and an analysis was performed to extract the codes, templates, and topics found in all participants' interview copies. In the second phase, we focused on data reduction and generation of the initial codes [8,17]. The data were encoded into important and easy-to-use pieces of text, such as excerpts, quotes, and individual words to develop specific themes. Based on the development of the initial topics and codes, we observed that the participants’ views converge almost on every topic [17]. As we repeatedly came across similar incidents, we empirically became aware that a category was saturated and stopped looking for groups and/or participants that extend the diversity of data [18]. Therefore, no further data collection and analysis was deemed necessary. In the third phase, we sorted codes into possible topics, classifying all data in the identified topics [8]. In the fourth phase, we focused on refining the design of the topics identified in the third phase, using a two-level analysis of the codes. In the fifth phase, we aimed at clearly identifying each issue and its meaning [8]. To achieve this, we focused on defining each topic, identifying its substance, and determining the aspect of the data and research queries appropriate for the topic in question [7]. In the sixth phase, we drafted the final analysis report.

The process described above produced the following six thematic axes: (1) Evaluation of primary health care, (2) evaluation of measures taken to streamline health expenditure, (3) affordability of health services, (4) personal experiences from the health care system, (5) confidence in the health care system, and (6) trust in health care professionals. For each of the six thematic axes, additional topics were created, and corresponding codes were given. Finally, we performed validity checks [19].

## Results

We interviewed and analyzed the responses of 50 participants but included the first 33 in this study because response saturation was observed at that point. Table 1 shows the demographics of the participants.

**Table 1:** Demographic data of the patients included in the analysis.

Demographics		
N	33	
Age	Mean: 54.3	(SD: 9.3)
Gender	Males	15
	Females	18
Marital status	Unmarried	10
	Married	19
	Divorced	3
	Widower	1
Annual income	0-15,000€	8
	15,001-25,000€	15
	>25,001€	10
Place of residence	Urban	8
	Suburb	20
	Countryside	5

Evaluation of primary health care

The analysis of the views of health care services user regarding primary health care is presented in Table 2.

**Table 2:** The responders’ opinion regarding health care services.

Opinion reported by the participants	Number of participants	%
Acknowledging the politeness and skills of the health care staff	25	75.75
Not noticing any changes	19	57.57
Identifying issues in the waiting areas of emergency rooms	17	51.51
Identifying an overall deficiency in the on-duty hospitals, reporting lack of staff, poor organization, crowded and problematic waiting rooms, and cleanliness issues	12	36.36
Identifying overall inadequacy of health centers in all sectors	10	30.30
Not expressing any opinion, never having visited a health center	10	30.30
Identifying staff shortages in health centers	6	18.18
Receiving immediate emergency services	5	15.15

Staff shortage, long waiting hours, dilapidated infrastructure, and lack of equipment and information were identified as the main issues. Key suggestions by the participants to improve the provided services included increasing staff, better facilities and equipment, providing easy access to medical services (shorter appointment dates), ensuring a humane approach-support of patients by medical staff, simplifying procedures, offering free health care, and creating new hospitals.

Additionally, the participants expressed their opinion regarding the electronic health record system, family doctor, and prevention, and the results are provided in Tables 3-5, respectively.

**Table 3:** The responders’ opinion regarding the electronic health record system.

Opinion reported by the participants	Number of participants	%
Being inadequately or not informed	19	57.57
Expressing concerns regarding the security of the electronic records	5	15.15
Recognizing its necessity, and its main advantage being that it facilitates the collection of their individual data with no need for extensive search	2	6.06

**Table 4:** The responders’ opinion regarding the family doctor.

Opinion reported by the participants	Number of participants	%
Not having insufficient information about the family doctor’s role	15	45.45
Not having registered a family doctor in their health record	15	45.45
Recognizing family doctor’s necessity	10	30.30
Identifying the large proportion of patients per doctor as a major problem	6	18.18
Being unaware of the registration procedure	5	15.15
Stating that the institution of the family doctor was not functional	3	9.09

**Table 5:** The responders’ opinion regarding the prevention.

Opinion reported by the participants	Number of participants	%
Stating that preventive initiatives were non-existent or were not aware of any kind of preventive initiative in mental health’s field	26	78.78
Acknowledging that adequate information on vaccines was provided	17	51.51
Acknowledging that significant steps had been taken toward free preventive examinations	15	45.45
Identifying initiatives regarding nutrition, smoking cessation, and dental hygiene	7	21.21
Reporting no change and inexistence of measures	6	18.18

Finally, regarding appointments, 18 (54.54%) participants reported unavailability of medical equipment, while six (18.18%) participants reported usually turning to private clinics. Finally, delayed appointments were considered the main disadvantage.

Evaluation of measures taken to streamline health expenditure

Table 6 shows the results of the analysis of the health care service users’ views regarding the measures for the rationalization of health expenditures.

**Table 6:** The responders’ opinion regarding the measures for the rationalization of health expenditures.

Opinion reported by the participants	Number of participants	%
Noticing changes regarding e-prescription	19	57.57
Having a positive opinion regarding e-prescription	13	39.39
Expressing trust in generic medicines	13	39.39
Identifying changes related to medication in general	12	36.36
Not having an opinion regarding generic medicines	7	21.21
Identifying changes related to generic medicines	7	21.21
Expressing concerns regarding generic medicines’ effectiveness and authenticity	5	15.15
Noticing changes regarding health campaigns	2	6.06

Regarding affordability of health services, users’ views on financial accessibility of health structures showed that the majority of participants could meet the cost of health care. Seven participants reported not being able to afford it, and two stated they made financial sacrifices to meet the cost. Twenty participants stated that the affordability depends on income and the amount of money each patient is willing to dispose. Five stated that they believe all health care services should be free.

Regarding the percentage of patient contribution in the payment of medication and check-ups, the analysis showed that 14 patients would prefer not to pay for it, while nine stated that the percentage paid by the patient is high. Only three found it reasonable. Thirteen participants did not express any opinion on the percentage covered by the state for check-ups and appointments in private clinics. Seven stated that this amount was reasonable, while eight reported it was insufficient.

Personal experiences from the health care system

The results after analyzing the patients’ opinions regarding their personal experience in health care establishments are shown in Table 7.

**Table 7:** The responders’ opinion regarding their personal experience in health care establishments.

Opinion reported by the participants	Number of participants	%
Reporting a lack of staff in public health centers	30	90.90
Not expressing any opinion	14	42.42
Expressing an overall negative view regarding health centers	10	30.30
Stating that the quality of the services provided in public hospitals varies depending on the hospital in question	7	21.21
Reporting negative personal experiences in health care establishments	6	18.18
Stating that the public health centers should play a greater role in the Greek health care system	2	6.06

The majority of patients are satisfied with the services of private doctors affiliated with the Greek health care system (charging the patient), while nine participants stated that the quality of services is heavily dependent on the amount of money the patient is able and willing to spend. Five patients had no personal experience, and six reported a difficulty in finding an available date for an appointment.

Regarding the local health units, 15 participants did not express any opinion or were unaware of the term. Some participants with relevant personal experience reported issues with the appointment dates, lack of staff and organization, and inadequate infrastructure.

Regarding confidence in the health care system and health care professionals, 23 participants reported a total lack of trust in the health care system, pointing toward the unsatisfactory infrastructure, lack of welfare state, insufficient funding, obsolete equipment, and dissipation of funds. A few participants commented on the health care professionals; most of them acknowledged the staff’s skills but reported a lack of trust in them and a slow service.

## Discussion

This qualitative study provides important insight into the views and experiences of health service users in Greece, in the context of the recent health care system reforms, using the thematic content analysis. Participants mainly highlighted the overall lack of substantial changes and trust in the health care system. Lack in staff, equipment, organization, and resources was identified, combined with small-scale corruption related to problematic health care access and affordability. Although e-prescription was positively evaluated, the family doctor institution did not seem to meet the needs of the health service users.

Several European Union countries, especially in central and eastern Europe, have adopted reforms strengthening primary care with measures such as improving primary care structures, introducing the institution of the family doctor, and enhancing the satisfaction of health care service users [20,21]. The results in many cases did not meet the original objectives of the reforms. The family doctor institution, for example, seemed to generate dissatisfaction among health care users, as people are used to, and often prefer, immediate access to more specialized care. Zielinski et al. argues that patients who are accustomed to easy access to special treatment in hospitals find it difficult to accept a new primary health care system in which the general practitioner is the primary caregiver [22]. Our results confirm these findings, since the lack of information on the registration procedure and the role of the family doctor, and the high number of patients allocated to each doctor, led to poor use of this primary health care service, regardless of the recognition of its necessity.

Studies focusing on health care user satisfaction report that the level of satisfaction may be related to previous expectations linked to personal experience of health care users. Thus, patients having lower expectations, without extensive knowledge of or interest in the provided services, may express greater satisfaction with the services than patients with higher expectations. This study questioned health service users accustomed to an affordable public health care system, characterized by a rather easy access prior to the economic crisis. Therefore, user expectations were high, explaining in part the strong dissatisfaction developed in the recent years.

Interestingly, studies have shown that health care users who have chosen to bribe the health care professionals may try to justify these payments by expressing greater satisfaction [23,24]. Our findings partly confirm this, since there were reports associating the health care services quality with the amount of extra money the patient is willing to pay.

Sarantopoulou argues that, in Greece, most of the health reforms not only did not limit the total health expenditures but were ultimately detrimental to public health care [25]. Additionally, a study on health inequalities after austerity in Greece shows that the proportion of low-income individuals reporting unmet medical needs owing to cost doubled between 2008 (7%) and 2013 (13.9%) [26]. Our results support these findings. Patients indicated a direct relation between income and access to public health care services. Moreover, participants reported the tendency to turn to the private sector because of the increasing lack of staff, equipment, and resources characterizing the public health care system.

Furthermore, we showed that although the access to several preventive examinations related to public health relatively increased, such improvements were inexistent in the mental health sector. Future studies are required to determine the importance of this issue and the ways to tackle it, especially in the context of COVID-19 and its repercussions on the mental health of the Greek population [27].

This study has several limitations. First, the sample was not geographically representative. Further research including a number of major Greek cities is required to confirm our findings. Second, thematic content analysis is a descriptive method. Additional methods are required to achieve a more complete analysis of research findings.

## Conclusions

Greece is currently implementing an ambitious reform plan to improve the efficiency of the health system and tackle important issues. However, patient experience shows that additional effort is required since the adopted reforms did not seem to directly benefit the health care system, either because they were not fully applied or because their implementation did not yield the expected results. Patients report that the persisting unequal availability of resources is a major problem, and the improvement of primary health care depends particularly on the availability of doctors. Adequate funding is also considered a prerequisite. Greece will benefit from a general and comprehensive reform plan that will take into account the health system’s overall performance and the needs of the population and will focus on proper planning and equal distribution of services.

## References

[1] Organisation for Economic Co-Operation and Development (2020). Anti-Corruption Network for Eastern Europe and Central Asia. Annual report.

[2] Tsountas G, Vardavas K, Giannopoulou Κ (2016). Greeks’ Health During Crisis [in Greek]. https://www.dianeosis.org/wp-content/uploads/2016/03/ygeia_singles_complete_ver02.pdf.

[3] Niakas D (2013). Greek economic crisis and health care reforms: correcting the wrong prescription. Int J Health Serv.

[4] Lincoln YS, Guba EG (1985). Naturalistic Inquiry. Naturalistic inquiry.

[5] Papageorgiou G (1998). Methods in Sociological Research [in Greek]. Gutenberg-Tipothito.

[6] Gibbs GR (2007). Thematic coding and categorizing. Analyzing Qualitative Data.

[7] Tsiolis G. (2018). Thematic analysis of quality data [in Greek]. Research Paths in Social Sciences. Theoretical - Methodological Contributions and Case Studies.

[8] Braun V, Clarke V (2012). Thematic analysis. APA Handbook of Research Methods in Psychology. Vol. 2. Research Designs: Quantitative, Qualitative, Neuropsychological, and Biological.

[9] Sherif M, Hovland CI (1961). Social Judgements: Assimilation and Contrast Effects in Communication and Attitude Change. Yale University Press.

[10] Thibaut JW, Kelley HH (1959). The Social Psychology of Groups. Wiley.

[11] Howard J, Sheth J (1969). The Theory of Buyer Behaviour.

[12] CA JM, AR E (1963). Some hedonic consequences of the confirmation and disconfirmation of expectancies. J Abnorm Soc Psychol.

[13] Oliver R (1976). Hedonic reactions to the disconfirmation of product performance expectations: some moderating conditions. J Appl Psychol.

[14] Cronin JJ Jr, Taylor SA (1994). Servperf versus Servqual: reconciling performance-based and perceptions-minus-expectations measurement of service quality. J Mark.

[15] Parasuraman A, Zeithaml VA, Berry L (1985). A conceptual model of service quality and its implications for future research. J Mark.

[16] Bazeley P, Jackson K (2013). Qualitative Data Analysis With NVivo. https://books.google.gr/books?hl=en&lr=&id=OGuPDwAAQBAJ&oi=fnd&pg=PP1&dq=Qualitative+data+analysis+with+NVivo&ots=1elx9mMYFs&sig=wCtZFsLs7v6qBvzmCprSl8tSLvA&redir_esc=y#v=onepage&q=Qualitative%20data%20analysis%20with%20NVivo&f=false.

[17] Attride-Stirling J (2001). Thematic networks: an analytic tool for qualitative research. Qual Res.

[18] Saunders B, Sim J, Kingstone T (2018). Saturation in qualitative research: exploring its conceptualization and operationalization. Qual Quant.

[19] Long T, Johnson M (2000). Rigour, reliability and validity in qualitative research. Clin Eff Nurs.

[20] Buciuniene I, Blazeviciene A, Bliudziute E (2005). Health care reform and job satisfaction of primary health care physicians in Lithuania. BMC Fam Pract.

[21] Bankauskaite V, Saarelma O (2003). Why are people dissatisfied with medical care services in Lithuania? A qualitative study using responses to open-ended questions. Int J Qual Health Care.

[22] Zielinski A, Håkansson A, Jurgutis A, Ovhed I, Halling A (2008). Differences in referral rates to specialised health care from four primary health care models in Klaipeda, Lithuania. BMC Fam Pract.

[23] Bleich SN, Ozaltin E, Murray CK (2009). How does satisfaction with the health-care system relate to patient experience?. Bull World Health Organ.

[24] Bjertnaes OA, Sjetne IS, Iversen HH (2012). Overall patient satisfaction with hospitals: effects of patient-reported experiences and fulfilment of expectations. BMJ Qual Saf.

[25] Sarantopoulou Z (2015). The positive and negative points of the reforms in the light of their contribution to the reduction of health expenditures from the beginning of the crisis until today [in Greek]. Scientific Chronicles.

[26] Karanikolos M, Kentikelenis A (2016). Health inequalities after austerity in Greece. Int J Equity Health.

[27] Giannopoulou I, Tsobanoglou GO (2020). COVID-19 pandemic: challenges and opportunities for the Greek health care system. Ir J Psychol Med.

